# A Rare Case of Zosteriform Cutaneous Metastases from a Nasopharyngeal Carcinoma

**DOI:** 10.1155/2015/415393

**Published:** 2015-11-30

**Authors:** Andrés González García, Emiliano Grillo Fernández, Ignacio Barbolla Díaz, Asunción Ballester, Héctor Pian, Guadalupe Fraile

**Affiliations:** ^1^Department of Internal Medicine, University Hospital Ramón y Cajal, Madrid, Spain; ^2^Department of Dermatology, University Hospital Ramón y Cajal, Madrid, Spain; ^3^Department of Pathology, University Hospital Ramón y Cajal, Madrid, Spain

## Abstract

From a clinical point of view, the most common presentations of cutaneous metastatic disease are papules and nodules. However, a wide morphological spectrum of lesions has been described, including erythematous patches or plaques, inflammatory erysipelas-like lesions, diffuse sclerodermiform lesions with induration of the skin, telangiectatic papulovesicles, purpuric plaques mimicking vasculitis, and alopecia areata like scalp lesions. The so-called zosteriform pattern has been described to be in few cases and to the best of our knowledge has never been described associated with a metastasis of a nasopharyngeal carcinoma. This case highlights the relevance of including cutaneous metastases in the differential diagnosis of patients with nonhealing herpes zoster-like lesions, especially in those with underlying neoplasm recently diagnosed.

## 1. Introduction

Nasopharyngeal carcinoma, although rare in western countries, is a common tumor arising in the nasopharyngeal region [1]. The lesions are mostly undifferentiated carcinomas and are associated with Epstein-Barr virus. On the other hand, the skin metastases are rarely reported [2]. Despite the infrequent skin involvement in these tumors, zosteriform pattern metastasis is a rare, not well-defined, entity, with only few cases published in literature in other kind of solid tumors [3].

We present a case of a zosteriform distribution of its cutaneous metastases, which has not been described previously.

## 2. Case Report

A 63-year-old man was referred for evaluation in Internal Medicine Department due to enlargement neck masses without any other symptomatology. Previously, he had been referred to an otorhinolaryngologist because of a mild hypoacusis. Several biopsies were undertaken from the neck masses and the histology revealed lesions with a necrotic background, a polymorphic lymphoid cell population, and poorly differentiated syncytial groups of atypical cells of epithelial lineage.

The complementary studies demonstrated osteolytic lesions in the vertebral column compatible with metastatic infiltration. At that moment there was no evidence of metastasis spread at any other location. A chemotherapy cycle was begun and provided a good clinical response.

**Figure 1:**
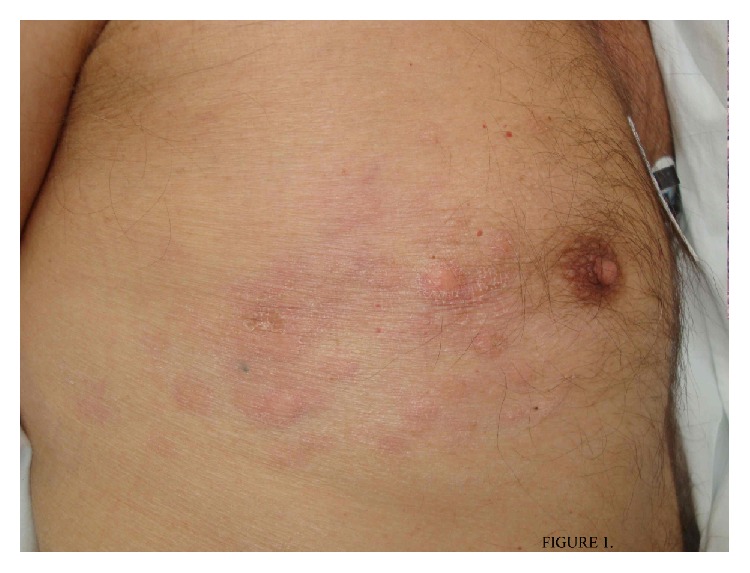
Erythematous nodules following a metameric distribution on the chest.

**Figure 2:**
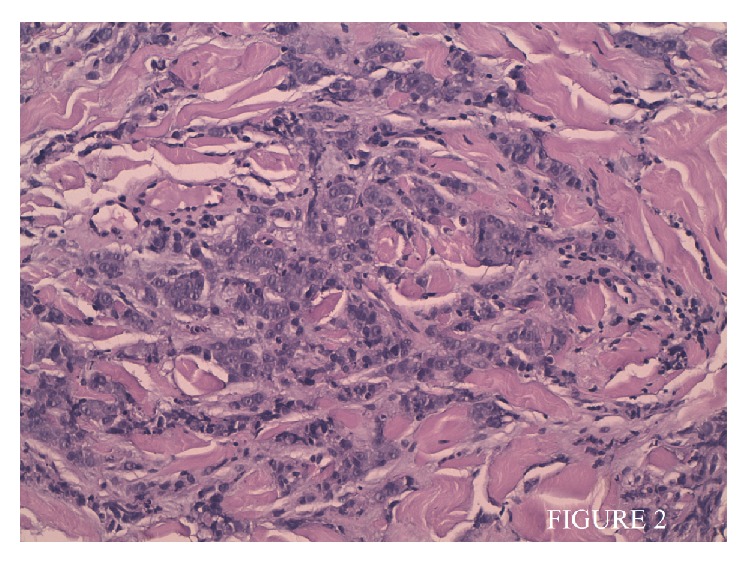
Infiltration of deep dermis syncytial groups of large cells with vacuolated clear cytoplasm and vesicular nuclei.

**Figure 3:**
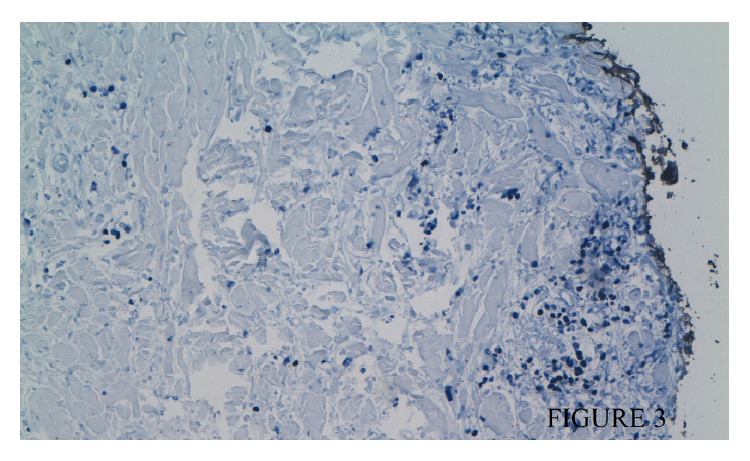
Dermis cell with Epstein-Barr virus- (EBV-) encoded RNA (EBER).

Three months later, the patient was admitted to the Emergency Department because he had presented with asthenia and painful erythematous, papule-nodular lesions in the left side of the chest. The temperature was 36,2°C, the pulse 82 beats per minute, the blood pressure 100/66 mm Hg, and the respiratory rate 18 breaths per minute. There were erythematous papules following a metameric distribution on the chest and several lesions in the posterior neck area, one of which adhered to deeper underlying structures (Figure 1). This eruption was treated as a herpes zoster infection by his family physician with 3 weeks of valacyclovir without improvement. With the patient's background in mind, a biopsy from the lesions on the chest was performed and revealed infiltration of a nonkeratinizing lymphoepithelioma-like lesion (Figures 2 and 3). The patient was transferred to the Oncology Department in order to begin with a proper treatment for its condition. Unfortunately new computed tomographic (CT) scans showed evidence of metastatic disease, with new bone lesions and lung nodules. After 3 weeks, the bone lesions had progressed and pain developed, and palliative irradiation and pain medication were required. One month later he opted to pursue palliative care only before he dies.

## 3. Discussion

Lymphoepithelioma-like carcinoma consists of a proliferation of poorly differentiated epithelial tumor cells with large vesicular nuclei and prominent nucleoli surrounded and infiltrated by dense lymphoplasmocytic infiltrates, mainly T lymphocytes. This tumor is a distinctive subtype of nasopharyngeal carcinoma but may also affect other organs such as the salivary gland, thymus, lungs, and skin [4].

Despite the well described skin involvement with solid tumors, zosteriform metastases are a rare entity, with only few cases published in the literature. A recent meta-analysis reviewed 4,774 patients published in the English literature since 1970 with zosteriform pattern cutaneous involvement. There were eight (14%) lymphomas (1 Hodgkin's lymphoma, 2 non-Hodgkin's lymphomas, 3 cutaneous B-cell lymphomas, and 2 cutaneous T-cell lymphomas), seven (12%) breast cancers, seven (12%) squamous cell carcinomas (SCC), six (11%) digestive tumors (2 gastric tumors, 3 colon tumors, and 1 gallbladder tumor), six (11%) respiratory tumors (5 lung tumors and 1 larynx tumor), and four (7%) urinary tumors (2 kidney tumors, 1 bladder tumor, and 1 prostate tumor) [3]. Many of the dermatomal metastases have been initially diagnosed as herpes zoster which is a common finding in immunocompromised oncology patients [5].

Several hypotheses tried to explain the mechanism of these patterns, but none of these theories have been validated. In our biopsy Epstein-Barr virus (EBV) genome was detected and its association with nasopharyngeal carcinoma and lymphoepithelioma variant is well known [6].

As both the principal etiology agent zoster-varicella virus (ZVV) and EBV belong to the Herpesviridae genre it is not unreasonable to hypothesize that EBV could contribute to the neurotropic distribution of the metastases. Watabe et al. described one case with a zosteriform pattern on a T-cell skin lymphoma where high EBV copies were detected. They gave more importance to the viral role as principal head of the dermatology distribution and the genesis of the tumor [7].

To the best of our knowledge, nasopharyngeal carcinoma has not been described to be associated with a metastatic zosteriform pattern. We performed a search of the medical literature by using the Embase, MEDLINE, and Scopus databases. We used the following terms as subject headings: “nasopharyngeal carcinoma,” “exanthema,” “skin neoplasms, secondary,” and “herpes zoster.” In addition, we searched the following keyword terms in all possible combinations: rash, zosteriform, papule, and nasopharyngeal. All references of each relevant report of the above searches were examined. The search disclosed no prior reports of nasopharyngeal carcinoma that caused a zosteriform rash.

## 4. Conclusions

Because dermatome metastases can mimic herpes zoster, which is a common finding in the immunocompromised oncology patient [3, 8] many of the cases in the literature were initially diagnosed and treated with antiviral agents without improvement. It is important to establish an elevated grade of suspicion in these patients in order not to delay the diagnosis, particularly when there is a background.

The case presented highlights the relevance of including cutaneous metastases in the differential diagnosis of patients with nonhealing herpes zoster-like lesions, especially in those with underlying neoplasm recently diagnosed.
